# Model selection and averaging in the assessment of the drivers of household food waste to reduce the probability of false positives

**DOI:** 10.1371/journal.pone.0192075

**Published:** 2018-02-01

**Authors:** Matthew James Grainger, Lusine Aramyan, Simone Piras, Thomas Edward Quested, Simone Righi, Marco Setti, Matteo Vittuari, Gavin Bruce Stewart

**Affiliations:** 1 School of Natural and Environmental Sciences, Newcastle University, Newcastle upon Tyne, United Kingdom; 2 Wageningen Economic Research, Wageningen, The Netherlands; 3 Department of Agricultural and Food Sciences, Alma Mater Studiorum - University of Bologna, Bologna, Italy; 4 Waste & Resources Action Programme (WRAP), Second Floor, Blenheim Court, Banbury, Oxfordshire, United Kingdom; Western Sydney University, AUSTRALIA

## Abstract

Food waste from households contributes the greatest proportion to total food waste in developed countries. Therefore, food waste reduction requires an understanding of the socio-economic (contextual and behavioural) factors that lead to its generation within the household. Addressing such a complex subject calls for sound methodological approaches that until now have been conditioned by the large number of factors involved in waste generation, by the lack of a recognised definition, and by limited available data. This work contributes to food waste generation literature by using one of the largest available datasets that includes data on the objective amount of avoidable household food waste, along with information on a series of socio-economic factors. In order to address one aspect of the complexity of the problem, machine learning algorithms (random forests and boruta) for variable selection integrated with linear modelling, model selection and averaging are implemented. Model selection addresses model structural uncertainty, which is not routinely considered in assessments of food waste in literature. The main drivers of food waste in the home selected in the most parsimonious models include household size, the presence of fussy eaters, employment status, home ownership status, and the local authority. Results, regardless of which variable set the models are run on, point toward large households as being a key target element for food waste reduction interventions.

## Introduction

Food waste has been drawing increasing scholarly attention due to the sizeable proportions it has assumed, and its socio-economic and moral implications ([1];after having been well below 100, the yearly number of scientific papers including the keyword “food waste” reached 100 in 2007, then grew to 219 in 2011, and peaked 722 in 2016 see https://www.scopus.com/). Its definition has been characterized by a lively debate that lead different national and international organizations to identify different boundaries emphasizing diverse elements characterizing the food waste issue [2–4]. In what follows, we define food waste as “avoidable food waste”, i.e. any “food and drink thrown away that was, at some point prior to disposal, edible” [5].

In developed countries, households are responsible for the relatively largest proportion of all food wasted [1–3, 6]. Indeed, economic development and urbanisation result in the adoption of lifestyles, working conditions and social dynamics typical of urban areas which, in turn, increase food waste in the downstream segments of the value chain (retail, food services, and households) [7]. Furthermore, developed countries differ from one another as for the food waste generated and the policies adopted to address this challenge. Cross-country differences in waste generating behaviours may also depend on habits embedded in the national culture [6, 8]. This holds although urbanisation and globalisation create increasingly homogeneous dietary and food waste patterns worldwide [8].

Some authors have detected geographical differences in the individual behaviours towards food waste among EU countries, due to factors such as per-capita income, and citizens’ perception of sustainability issues [7, 9–11]. Although developed countries present high per-capita incomes, hunger in these countries is a reality: e.g., approximately 4% of US households, and more than 5% of Australian households are experiencing hunger [12]. On the other hand, the abundance of food results in high food waste levels [13]. For example, household food waste account for 6.7 million tonnes of edible food or 33% of all food purchased in the UK, 6.3 million tonnes or 20% of food purchased in Australia, and more than 160 million tonnes in the US [12]. If redistributed to people in need, this food could help reduce hunger, while food waste levels could also decrease.

Food waste generates negative environmental impacts and economic costs. It has been estimated that nearly one third of the food mass, and one quarter of the food calories globally produced are either lost or wasted, corresponding to 3.3 Gtonnes of CO_2_ equivalent [2, 7]. In the EU-28, annual food waste amounts to 180 kilograms per person, i.e. 25% of the food purchased by households [2,7]. Hence, the valorisation of physiological and unavoidable waste and residues as inputs for diverse productive processes, such as bioenergy or the production of bio-based products, might create socio-economic benefits and reduce environmental repercussions. Indeed, most wasted foods are of primary interest to biofuel production [13]. Nevertheless, the social, economic and environmental viability of food waste as a source of biofuel remains underdeveloped, thus requiring effective strategies to reduce food waste generation.

Overall, food waste is a broad topic that has been discussed from different angles in recent literature [1–3, 6, 12, 13]. In order to contribute to the understanding of and the reduction of food waste, various attempts have been made to identify and analyse the socio-demographic factors influencing food waste behaviours (e.g. [7, 14–22]). This study focuses on the drivers of food waste generation at household level. Indeed, a proper identification of them could help design effective policies for food waste prevention and reduction.

The rest of the paper is organised as follows. The next section provides a background on food waste drivers at household level, and addresses the issues of complexity related to the empirical modelling of these drivers. A detailed description of the data and methodology can be found in the subsequent section, followed by the results and their discussion.

### Food waste drivers at household level

Individual and situational factors leading to the generation of household food waste include household characteristics, shopping habits and location in relation to shops, eating/cooking behaviours, and awareness (e.g. the understanding of date labels on products, attitudes to waste and recycling, consumer preference for perceived high-quality food, etc.).

Literature suggests that food waste is influenced by household characteristics with a major factor represented by the composition of the family:

- in absolute terms, larger households waste more food than smaller households, but they are also more efficient, wasting less food per person than smaller households; instead, single-person households tend to throw away more food on a per capita basis [6, 20–25];
- adults waste more in absolute terms than children, but households with children tend to waste more than households without children, with food waste rates varying with children’s age [6, 20–25];
- the gender of the person mainly responsible for grocery shopping, and for food storing and cooking might also have implications [6, 7, 20, 24];
- differences between older and younger people are not consistent, yet retired households seem to waste less because they have more available time (compared to younger households and households with children) and tend to be smaller [6, 20–23, 26, 27];
- income levels matter, but the relationship between individual income, food behaviours, and household food waste [10, 28, 29].

As for shopping habits, the frequency of shopping [20, 30–32], the location of the stores related to the frequency of the purchase, and the planning of the shopping [24, 33] represent other aspects of consumer behaviour related to food waste. On the one hand, consumers may over-purchase if they need to shop infrequently [24, 33]; on the other hand, frequent shopping may induce unplanned and impulsive purchases, which tend to increase food waste [34]. Not planning shopping trips, absence of shopping lists, not planning meals, and not checking stocks lead to the generation of food waste at household level [3, 7, 35–39].

Lack of awareness and/or knowledge is one of the most commonly identified drivers of food waste at household level [2, 10, 21–23, 30, 32, 37, 40]. This includes consumers’ confusion with product labelling, as well as a lack of knowledge on how to use food efficiently—e.g. making the most of leftovers, or cooking with available ingredients [37, 38]. Consumers are rarely aware of the difference between the labels “use by” and “best before”; hence, they are not using them effectively when planning food usage and/or discard to avoid the risks associated to food safety [3, 37, 38, 41, 42]. Not understanding and/or not abiding by food storage and use instructions provided on food packages also leads to food waste [42]. Finally, consumers may not use packaging functionality, e.g. taking some products out of their packaging after getting home, thus losing the protection of modified atmosphere packaging, or not using cool bags to bring chilled food home [33, 35, 43, 44].

While food waste drivers have been discussed extensively in recent literature, their relative importance and their interactions have received little attention. Literature suggests that food waste drivers are multiple and interrelated, characterizing the problem as “wide and multifaceted” [7]. This framework is further complicated by the time and location gap “between choices made upstream (food purchasing and using decisions) and actions downstream (frequency of household food waste)”, which prevents intentionality and commitment from working effectively [10].

Besides, since different authors propose different definitions of food waste, the boundaries of the systems considered are also different (e.g., what is avoidable and non-avoidable food waste) [4]. This lack of consistency in the notion of food waste may lead consumers to resort to their subjective perception of what food waste is, when asked to assess related behaviours and quantities. Indeed, the adoption of different methodologies for data collection (questionnaires, diaries, waste sorting analysis), or of poor or no measurements hampers the lack of consistency in terms of quantification [4, 45]. Due to the high costs of measuring household food waste, most studies in the existing literature base their inference on self-reported measures detected by means of questionnaires. Here, the use of real food waste as a dependent variable helps overcome the problem of underestimation for social desirability bias, and of misreporting due to other behavioural biases, thus reducing the risk of incorrect inference.

### Addressing complexity in food waste models

The high number of interconnected food waste drivers described above implies that traditional modelling approaches may not be appropriate, or need specific adjustments. The approaches to address multivariate problems have traditionally followed a procedure whereby data are collected on several variables that may plausibly explain the response variable, and analysed to find a single “best” model [46]. The model’s structure is often defined *a priori*, and the estimate from this model then forms the basis of inference. This approach ignores the potential for other models to explain the data, and this model uncertainty increases the potential for incorrect or misleading inference [47]. This is shown empirically for sociological models (OLE regression) by Young [48], where statistical significance is overturned by minor and sensible changes in model structure. Hence, there is a higher probability of false inferences (i.e. Type I errors or false positives, and Type II errors or false negatives).

False positives (Type I) are often more costly than false negatives (Type II) because they lead to wasted resources on further research and ineffective policy interventions [49]. The probability of Type I errors can be increased by increasing the number of parameters modelled but also by “researcher degrees of freedom” (*sensu* [49]). Unreported aspects of the research can lead to increased risk of false positives through changes in the selection of dependent variables or covariates, altering sample sizes and only reporting subsets of experimental conditions [49].

Food waste drivers are multiple, interconnected and influenced by a number of diverse factors related to the influence of the technological, institutional and social “contexts” where they are situated [7]. Addressing such a complexity requires the inclusion of multiple explanatory variables, increasing the risk of Type I and Type II errors. However, most assessments of food waste use a regression framework with multiple explanatory variables without addressing issues of model structural uncertainty, and rely on a single model specification, based either on the extant literature or on the author’s hypotheses, to make inferences from (e.g. [9, 10, 32, 50, 51]). Basically, while the set of variables gathered are bounded to be selected according to the theory of the collectors, it is possible to avoid any further bias on the model construction due to the artificial selection of variables and interaction terms to be included in the model itself. This theory-based approach (using one single model) is blinkered to other possible explanatory models (within the realms of the data collected). In presence of multiple potential explanatory variables, model selection has long been championed as being more robust to Type I errors [52].

Here, we adopt a novel empirical approach to identifying the drivers of food waste to inform waste reduction policies. Our approaches for variable and model selection, differ from the more common (and highly biased; [53]) stepwise selection based on the coefficients’ level of significance. With this approach, the aim is to identify the key drivers of household food waste, whilst more accurately reflecting the uncertainty inherent in the analysis of observational multidimensional data.

## Materials and methods

### Data

Data on UK consumers’ demographics and behaviours collected by The Waste and Resources Action Programme [5] are used in order to appraise the weight of “avoidable food waste per household” using model selection and model averaging [54] to account for model uncertainty.

The dataset consists of face-to-face in-home interview responses (categorical data) on socio-demographic aspects of households and behavioural responses to food waste, along with data on the amount of waste collected from the kerbside. We undertook a complete case analysis utilising only the households for whom all information was reported, which resulted in a sample size of 1,770 (from 1,799) UK households. Household waste was collected from outside each home (flats and houses with shared waste collections were not assessed) by ad hoc teams. After collection, the waste of each household was weighed and sorted. All non-food items were removed and weighed. Food items without packaging were sorted by food type and then weighed. Food items with packaging were removed from the packaging, weighed separately, and any details on the packaging (e.g. best before dates) were recorded (for more details, see [5] and references within). Finally, food waste was standardised per household (i.e. food waste per person was calculated) to account for the difference that a larger number of family members could make to the amount of waste produced.

### Variable reduction

With 50 variables, the set of potential models was well over a quadrillion and, therefore, variable reduction was first undertaken using the random forest algorithm [55]. The “Boruta” algorithm (in the package “Boruta”, [56], in the R statistical environment [57]; all R code for analyses is provided in S1 File) adds randomness to the variable set by creating shuffled copies of all variables (“shadow features”). It then runs a random forest classifier on the extended dataset, and assesses the mean decrease in accuracy to evaluate the importance of each variable (higher means are more important). At each iteration, “Boruta” assesses if each variable has a higher Z-score than the maximum Z-score of its shadow features. Variables with scores lower than shadow features are deemed highly unimportant, and removed from the set. The algorithm runs until all variables are confirmed or rejected (or it reaches a specified limit of runs—here, we used 500 trees maximum).

### Modelling

Generalised Linear Models (GLMs) were applied to assess correlations between “avoidable household food waste” and the socio-demographic and behavioural variables retained after applying the “Boruta” algorithm (Table 1).

**Table 1:** The variables used in the development of regression models assessing the drivers of consumer food waste (note that some variables listed below are multifaceted due to the various product types addressed). Avoidable food waste was the dependent variable and the others were the explanatory variables.

Variable	Definition	Measurement
Avoidable Food waste	Food and drink thrown away that was, at some point prior to disposal, edible, e.g. milk, lettuce, fruit juice, meat (excluding bones, skin, etc.)	Weight (g)
		Minimum: 0
		1st Quartile: 379
		Median: 1080
		Mean: 1668
		3rd Quartile: 2300
		Maximum:19836
Gender	Sex of the person responsible for the majority of the household shopping and cooking	Male/Female
Age structure	Based on ages of all household members	Mixed aged household
		Under 65 years old only
		65 years and above only
Household size	The number of people in the household	1,2,3,4,5,or 6 people
Household composition	Description of the household composition	Couple
		Family with at least one child under 18 years olds
		Family with child(ren) all 18 years or over
		Single occupancy
		Other
Home ownership	The ownership status of the house, e.g. privately rented or owned with mortgage	Council/housing association rented
		Owned outright
		Owned with a mortgage
		Privately rented
		Other
Type of residence,	The type of house lived in	Bungalow
		Detached house
		Semi-detached house
		Terraced house
		Flat
		Other
Presence of children	Are children between 3 & 11 years present in the household	Yes/No
Social-economic status	Calculated based on the characteristics of the main earner	ABC1: Higher & intermediate managerial, administrative, professional occupations or Supervisory, clerical & junior managerial, administrative, professional occupations
		C2: Skilled manual occupations
		DE: Semi-skilled & unskilled manual occupations, Unemployed and lowest grade occupations
Employment status	The employment status of the person responsible for the majority of the household shopping and cooking	Not working Paid work Retired Not stated
Pre planning	The extent to which meals are planned	All main meals are planned
		Most main meals are planned
		Few main meals are planned
		Decide on the day
Cupboard checking	Are the cupboards/Fridge/Freezer checked before shopping trips for:	Yes
	Fruit	No
	Vegetables	Don’t Know
	Bread	Don’t buy the item
	Meat	
	Fish	
	Milk	
	Ready meals	
	Tinned food	
	Frozen food	
	Salads	
Preplanning list	Do the statements describe pre-shopping behaviours?	Yes/No
	Kept a ‘running list’ during the week of things needed to buy	
	I made a list to take to the shop with me	
	I had a very clear list in my head	
	I had some idea of the kind of things I wanted to buy	
	I shopped online, and I used my list of favourites to help me remember what to buy	
	None of the above	
	Don’t know/can’t remember	
	Not stated	
Storage of cheese and meats after opening	How cheese and meats are stored in the home	Don’t eat this food
		Wrapped / box / bag
		Original packaging
		No wrapping
		Other / don’t know
Use of the fridge to store apples and carrots	How apples and carrots are stored	Don’t buy / store
		Use fridge (and possible other place to store)
		Use fruit bowl/Use cupboard
		Other storage
		Don’t know / can’t remember
Using leftovers	What happened to the last left-overs from a meal	Yes
	Used as part of another meal	No
	no Used as a meal in themselves	
	Used as a meal in themselves	
	Didn’t get used and were thrown away	
	Still being stored	
	Composted	
	Fed to the dog/pets/birds	
	Given to family/friends	
	Never have left-overs	
	Placed in freezer/fridge/frozen for later use	
	Other	
	Don’t know/can’t remember	
	Not applicable	
	Not stated	
Cooking the right amount of rice and pasta	Was there rice or pasta left after a meal	Yes
		No
	Was too much cooked deliberately for use in another meal	Yes
		No
		Don’t Know
		Not Applicable
Throwing away items because they have gone past their date label	How much of the items list had been thrown away because they are past the date on their label:	Quite a lot
	Fresh meat	A reasonable amount
	Pre-cooked meats	A small amount
	Milk	Hardly any
	Yoghurts	None
	Ready meals	Don’t eat it
	Fruit juices	
	Bread or other bakery items	
	Fresh fruit	
	Vegetables	
	Frozen items	
	Any other items	
	None of the above	
	Don’t know/can’t remember	
	Not stated	
Type of shopping trips made	Shopping trip types	I buy almost all my food in a main shop
		I buy some food in a main shopping trip and some in ‘top-up’ shops
		I mostly buy food in smaller, ‘top-up’ shops
		Not stated
Frequency of main shopping trip	Shopping habits	I do a main shop more than once a week
		I do a main shop about once a week
		I do a main shop about once a fortnight
		I do a main shop about once a month
		I almost never do a main shop
Fussy eaters	Proportion of occupants of the household classed by the survey respondent to be fussy eaters	Proportion (between 0 and 1)

All categorical variables were treated as factors in the analysis. The Akaike Information Criterion corrected for small sample size (AICc) was used to determine a set of plausible models; modelling averaging [54] was used to obtain estimates of the effect of predictors on “avoidable household food waste”. Variables that were retained in the model selection procedure were assessed for interaction. GLMs, model selection and model averaging were carried out using the “glmulti” package [58] in the R programme.

### Exploratory sensitivity analysis

The variables summarizing the self-reported discard of different types of food have the potential to introduce circularity, as they may predict overall food waste. Therefore, after running the variable reduction and model-selection procedures, we removed them from the full model, and re-ran these two steps. Similarly, the local authority was considered as a non-designed confounder (it was recorded but without any underlying justification). Again, we removed this variable in the full model and re-ran the analysis. Finally, we re-ran the analysis with both discard behaviours and local authority removed.

## Results

### Model set reduction

**Fig 1:**
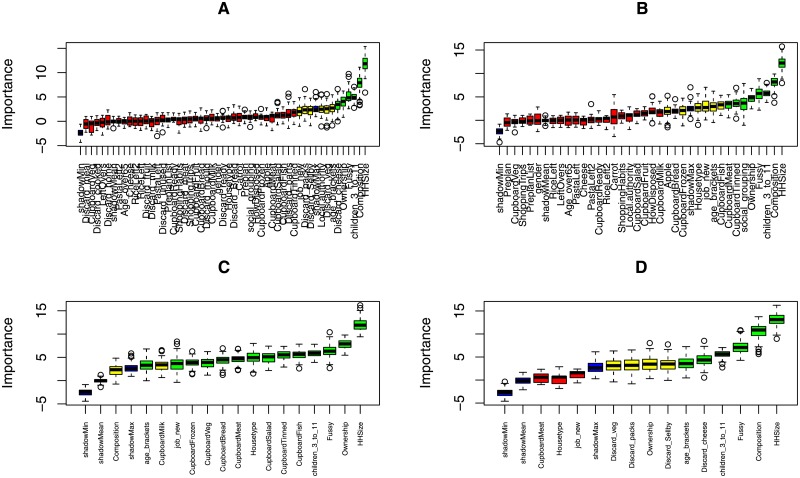
a) Variable importance (the loss of accuracy of classification) as determined by the “Boruta” algorithm for the full variable set. Variables retained for model selection (those with high or medium importance) are highlighted in green and yellow. Shadow feature minimum, mean and maximum are highlighted in blue; b) with “Discard behaviours” removed from the variable set; c) with “Local authority” removed from the variable set; d) with both “local authority” and “discard behaviours” removed from the variable set.

The “Boruta” algorithm consistently identified household size, home ownership status, household composition, employment status and the presence of fussy eaters as significant drivers of food waste in all sets of variables (Fig 1a–1d), including those reduced for exploratory sensitivity analysis. Household size was always the most important variable in the variable set (Fig 1a–1d).

### Model selection

The key drivers of consumers food waste included in the full model (as determined by the “Boruta” algorithm, Fig 1a) were household size, local authority, household composition, house type, home ownership status, employment status, the presence of fussy eaters, the presence of children aged between 3 and 11, age of the respondent, social grouping, checking cupboards for tinned food prior to shopping, and discard behaviours related to vegetables, cheese, and food past its sell by date. This equated to a potential 16,384 models.

Of the 14 variables, seven were retained in the final model sets (the most parsimonious models, ΔAICc <2; see Table 2).

**Table 2:** Five plausible models (ΔAIC <2.0) were selected from the original set of 16,384 models. Models were ranked by AICc (“:” indicates interaction terms). The averaged coefficients of the models are shown in S1 Table.

Component models:	df	logLik	AICc	delta	weight
Avoidable waste per Household ~ Discard Sellby+ Discard vegetables+ Fussy eaters + Household size + employment+ Local Authority + Home ownership+ Discard Vegetables:Fussy eaters+ Fussy eaters:Employment	47	-15481.2	31059.1	0	0.28
Avoidable waste per Household ~ Age+ Discard Sellby+ Discard vegetables+ Fussy eaters + Household size + employment+ Local Authority + Home ownership+ Discard Vegetables:Fussy eaters+ Fussy eaters:Employment	48	-15480.2	31059.13	0.03	0.28
Avoidable waste per Household ~ Age+ Discard Sellby+ Discard vegetables+ Fussy eaters + Household size + employment+ Local Authority +Discard Vegetables:Fussy eaters+ Fussy eaters:Employment	44	-15484.9	31060.1	1	0.17
Avoidable waste per Household ~ Age+ Discard vegetables+ Fussy eaters + Household size + employment+ Local Authority + Home ownership+ Discard Vegetables:Fussy eaters+ Fussy eaters:Employment	42	-15487.1	31060.39	1.29	0.15
Avoidable waste per Household ~ Sellby+ Discard vegetables+ Fussy eaters + Household size + employment+ Local Authority + Home ownership+ Discard Vegetables:Fussy eaters+ Fussy eaters:Employment	41	-15488.4	31060.78	1.68	0.12

The variables with the largest positive effect included the presence of fussy eaters, household size, and one particular local authority (individual local authority identity was anonymized). Variables with the largest negative effect included discard behaviours interacting with the presence of fussy eaters, employment status interacting with the presence of fussy eaters, four specific local authorities and home ownership status (owning a house outright).

### Exploratory sensitivity analysis

The variables included in the model with discard behaviours removed (Fig 1b) were household size, local authority, household composition, house type, home ownership status, the presence of fussy eaters, and employment status. This equated to a potential 128 models. The final model set included six of these variables: household size, local authority, home ownership status, the presence of fussy eaters, respondent age, and employment status (S2 Table). Variables with the largest positive effect included the presence of fussy eaters, employment (working), household size (increasing with a larger number of occupants) and age (35–64). Variables with the largest negative effect included interactions between fussy eaters and employment, age (35–64), employment (not working), two specific local authorities, and home ownership status (with a mortgage or owned outright).

Variables with the largest positive effect in the model with local authority removed (see Fig 1c for the variables retained) included household size (two, three, four or five people), while variables with the largest negative effect included home ownership (owned outright and owned with a mortgage) and employment (retired) (See S3 Table). The results of the models with both local authority and discard behaviours excluded were very similar (See S4 Table).

## Discussion

### The drivers of UK household food waste

The variables selected in the most parsimonious models always included household size, the presence of fussy eaters, employment, home ownership status, and local authority. Household size (i.e. the number of people in the household) appears to be a generally well-supported explanatory variable [14, 16–18, 20–22, 59]. Levels of avoidable food waste per household increased with increasing household size. Aschermann-Witzel et al. [60] suggest that household size and composition (i.e. the age of household members) are the key demographic drivers of food waste, because they relate to multiple behavioural factors, which typically differ across household types. These include, for example, the purported advanced food skills of the older generation (making use of leftovers, etc.), higher food security and safety concerns of households with children, greater levels of fussiness in households with children, and lower degrees of planning in young or single-person households. Our results support the idea that fussiness in a household has a small but noteworthy effect on food waste generation.

Regardless of variable set, our results point toward families (i.e. large households) as being a key target group for food waste reduction initiatives. Targeted initiatives (such as educational campaigns and increased frequency and modalities of waste collection) in areas with a high density of larger households need to be prioritised for study and intervention. Other evidence [61] indicates that the reasons these households waste food are more likely to be due to cooking or serving too much or fussy eating (rather than not using food before it goes off).

Survey respondents stating that they discard “a reasonable amount” of vegetables was related to higher levels of waste compared to other food categories. Discarding “quite a lot” had a similar mean value of the remaining food categories, but greater variation. Low levels of vegetables discarded by consumers logically lead to reduced avoidable food waste as vegetables are the single largest food group contributing to household food waste in the UK [5]. However, there may be some discrepancy between stated and actual levels of discard due to a range of factors [62]. Interventions aimed at preventing vegetable waste through, for example, supporting the purchase of an appropriate amount, storing it optimally or providing recipes to help use up leftovers may help further reduce food waste.

Local authority was not intended as a predictive variable in the original data collection, as there were no socio-demographic assumptions underlying the sampling regime. The fact that this descriptive variable (treated as a random variable in the model) is an important explanatory variable highlights the large geographical variability in the food waste behaviours observed. A combination of imprecision and high heterogeneity in the variables used to assess consumer food waste may explain the difficulty in determining significant relationships. An alternative explanation is that regional factors are important (but we could not determine any evidence for this in our dataset). The location could be a proxy for socio-economic factors, as well as factors related to the availability and the identity of retailers. Further investigation into the drivers of these regional differences is warranted.

### Developing an evidence-based approach to food waste

By using model selection to identify the most suitable structure of a model, researchers can reduce the probability of spurious results. The danger of Type I errors is that they lead to increased uncertainty in the effectiveness of interventions, because of both incorrectly targeting consumers’ behaviours and wrongly assigning significance to specific interventions. Selective reporting, where only some of the variables measured are reported in the outcome, further reduces the ability to synthesise across studies (e.g. through systematic review and meta-analysis) an issue already highlighted as a constraint in consumer food waste research [63].

Type II errors are reduced effectively by increasing the sample size; however, Type I errors may still be highly probable where a large number of variables are used (i.e. “p-hacking”), and/or where many models are run but only those which confirm pre-conceived ideas or theories are reported. To effectively reduce Type I error (one can never totally eliminate Type I or Type II errors), researchers can take a number of potential approaches:

1. Careful selection of variables with a rationale for inclusion: a pre-published protocol can be used to identify the variables that will be tested and processed to reduce the biases undertaken by the researcher. This is a popular approach in meta-analysis and systematic review, but can be applied more widely.
2. Provision of all analysis and data in the rawest possible form in an open online data repository (e.g. Open Science Foundation, https://osf.io) to allow independent analysis (data sharing is not always appropriate or possible, due to commercial sensitivities, etc.).
3. Transparent variable selection and model averaging, as well as reporting multiple model results with a clear indication of the range of potential outcomes and the errors associated with these (e.g. confidence limits, credible intervals, etc.) should be standard practice.

Our approach accounts for model structural uncertainty in a frequentist paradigm. Of course, the issue of Type I errors becomes irrelevant when using Bayesian models, however with frequentist statistics still dominating research in consumer science there is a need to reduce the probability of spurious results in a robust manner. Stepwise approaches (which are superficially similar to our approach) have largely been discredited in many fields (e.g. in medicine and ecology; [53, 64]) because they increase (among other problems) the Type I error rate.

In addition to the problems of variable choice and Type I errors in models of consumer research, there are problems with the typical approaches to complexity adopted in this field. There is a well-developed body of complexity theory (e.g. [65]) which appears to be largely ignored in favour of a generic mixed methods approach to data acquisition and regression based modelling (e.g. factor analysis, structural equation modelling, mixed regression model, etc.). The lack of a coherent framework is often justified with adoption of a single theoretical perspective exacerbated by failure to consider model (structural) uncertainty. The tools to undertake more structured and nuanced analysis exist (e.g. agent based models, network analysis, systems dynamics; [66]) and should be routinely deployed in consumer research as they are in other scientific disciplines.

## Conclusions

The drivers of food waste are complex and interrelated, and may not lend themselves well to traditional modelling approaches. This high complexity may be better analysed through other statistical models or paradigms—such as Bayesian analysis—in order to reduce the probability of false positives. What is clear is that food waste policies must be developed using an evidence-based approach, since traditional modelling paradigms are not sufficient to address this complexity. This field of study can learn much from medicine and ecology, where data are often similarly complex and uncertain [67]. Standard protocols for data collection and definition would need to be agreed to allow meta-analysis. For data collection, protocols are emerging, such as the FUSIONS Definitional Framework for Food Waste [4] and Food Waste Quantification Manual [68] and the World Resources Institute *Food Loss and Waste Standard* (http://flwprotocol.org/). With more rigorous evidence-based approaches, the drivers of food waste can better be determined, and the effectiveness of any trialled intervention can be more certain. This will lead to decreased cost and a more meaningful contribution to the understanding of food waste.

Among the most important drivers identified is household size; however, the procedure of model reduction and selection allows us to uncover a positive relationship between household size and food waste, at odds with most of the previous literature on the issue [14, 23, 26, 69]. Other important drivers are the various dimensions of the household composition, for which the results corroborate those of the literature. Interestingly, some of the drivers identified as important by the literature, such as awareness of the food waste problem and shopping habits, here are found as not important. This testifies the relevance of unbiased model selection of an evidence-based approach to data analysis.

Finally, no evidence emerges on the behavioural characteristics of individuals at the point of purchase (i.e. in the supermarket), and on how they may influence the food waste generation. Any further research and, in particular, those focusing on large households, would need to include this aspect.

## Supporting information

S1 File The complete R Code. (DOCX)

S1 Table Model average coefficients for the full model including interaction terms. The variables are sorted by z value.
(DOCX)

S2 Table Selected models and averaged coefficients (sorted by z value) for the model with discard behaviour variables excluded. (DOCX)

S3 Table Selected models and averaged coefficients (sorted by z value) for the model with local authority excluded. (DOCX)

S4 Table Selected models and averaged coefficients (sorted by z value) for the model with local authority and discard behaviours excluded. (DOCX)
