# Supplementary material for: Model selection and averaging in the assessment of the drivers of household food waste to reduce the probability of false positives
Source: PLoS One. 2018 Feb 1;13(2):e0192075. doi: 10.1371/journal.pone.0192075 (PMC5794155; doi:10.1371/journal.pone.0192075)
Supplement: S1 File — (DOCX) [file pone.0192075.s001.docx]

**S1 File. The complete R Code**

*#THIS IS THE R CODE FOR THE ANALYSIS ON GRAINGER ET AL.*

*#Addressing complexity in the assessment of the drivers of consumer food waste*

*#matthew.grainger@ncl.ac.uk*

*#Set working directory*

setwd("C:/Users/Matt/Documents/REFRESH")

*#load packages*

**library**(ggplot2)

**library**(randomForest)

**library**(glmulti)

**library**(MuMIn)

options(na.action = "na.fail")

**library**(car)

*#Get data*

data<-read.csv("Data_Grainger_et_al_Type1error.csv",header=**TRUE**)

head(data)

names(data)

dim(data)

data<-data[which(data$Study==1),]

data<-data[which(data$IsNA.==0),]

dim(data)

lapply(data, **function**(x) {

**if** (is.numeric(x)) **return**(summary(x))

**if** (is.factor(x)) **return**(table(x))

})

*#remove serial number, Study & is.na columns*

data<-data[,2:55]

dim(data)

*#remove avoidable_FW & HH_size 2 columns*

data$Avoidable_FW<-**NULL**

data$HH_size_2<-**NULL**

dim(data)

str(data)

data$HHSize<-as.factor(data$HHSize)

summary(data)

*#######*

*#Full linear model*

*#How many models?*

glmulti(A_per_HH~.,data=data,level=1, method='d',confsetsize=10,plotty=T)

*#Error too many predictors*

glmulti(A_per_HH~.,data=data,level=2, method='d',confsetsize=10,plotty=T)*#with interaction*

*#Error Oversized candidate set*

mod1<-glm(A_per_HH~.,data=data)

sink(file="C:/Users/Matt/Documents/REFRESH/sink.txt")

summary(mod1)

sink()

*#######variable reduction using random forest*

*#discard cooked meat - more than 50 potential categories*

drops <- c("CookMeat")

data<-data[ , !(names(data) %in% drops)]

*#install.packages("party")*

*# library(party)*

*# cforestImpPlot <- function(x) {*

*# cforest_importance <<- v <- varimp(x)*

*# dotchart(v[order(v)],xlab="Permutation importance")*

*# }*

*# cf1 <- cforest(A_per_HH ~ ., data=data,control=cforest_unbiased())*

*# cforestImpPlot(cf1)*

*# install.packages("Boruta")*

**library**(Boruta)

Boruta1 <- Boruta(A_per_HH ~ ., data=data, doTrace = 2, ntree = 500)

print(Boruta1)

plot(Boruta1)

plot(Boruta1, xlab = "", xaxt = "n")

lz<-lapply(1:ncol(Boruta1$ImpHistory),**function**(i)

Boruta1$ImpHistory[is.finite(Boruta1$ImpHistory[,i]),i])

names(lz) <- colnames(Boruta1$ImpHistory)

Labels <- sort(sapply(lz,median))

axis(side = 1,las=2,labels = names(Labels),

at = 1:ncol(Boruta1$ImpHistory), cex.axis = 0.55)

*#selected variables*

myvars<-c("A_per_HH","Local.authority", "HHSize", "Q33_Recoded",

"Discard_veg", "job_new", "Q31_Recoded","Fussy","Discard_cheese","age_brackets",

"Discard_Sellby", "social_grouping", "children_3_to_11","Q36_Recoded",

"CupboardTinned")

data1<-data[myvars]

names(data1)

*###look at model set potential*

glmulti(A_per_HH~.,data=data1,level=1, method='d',confsetsize=100,plotty=T)

*# 16384 models*

glmulti(A_per_HH~.,data=data1,level=2, method='d',confsetsize=100,plotty=T)

*#1e9 models*

fit<-glm(A_per_HH~.,data=data1)

*#model selection*

dd<-dredge(fit)

ds<-subset(dd,delta<2)

final<-model.avg(ds)

sink(file="C:/Users/Matt/Documents/REFRESH/sink.txt")

summary(final)

sink()

*###*

*###Get full distribution of p*

modAll<-glmulti(A_per_HH~.,data=data1,level=1, method='h',confsetsize=100,plotty=F)

tbl<-weightable(modAll)

nrows <- nrow(tbl)

sink("ModelPvalues.txt",type="output")

**for** (i **in** 1:nrows) {

formulaTest <- as.character(tbl[i,]$model)

model = glm(as.formula(formulaTest), data=data1)

print(paste("Formula: ", formulaTest))

print(summary(model)$coefficients[,4])

}

sink()

*#need to adjust table txt in excel*

pdat<-read.csv("ModelPValues.csv",header=T)

head(pdat)

str(pdat)

graph1 <- **function**(x) {

ggplot(x, aes(x=p)) + geom_density(fill="red") + xlim(c(0,1))

}

graph1(pdat[pdat$Variable=='social_groupingDE',])

p<-ggplot(pdat, aes(x=p)) + geom_density(aes(fill="red"),alpha=.1)+

xlim(0,1)

p <- p + facet_wrap( ~Variable)

p

*# Generate ggplot2 plot for each level*

plot.list <- by(data = pdat,

INDICES = pdat$Variable,

simplify = **TRUE**,

FUN = **function**(x) {

p %+% x

}

)

**library**(ggplus)

**library**(gridExtra)

*# Build the grobs*

multi.plot <- marrangeGrob(grobs = plot.list,

nrow = 2, ncol = 2,

top = quote(paste('\nPage', g, 'of', pages)))

*# Plot on multiple pages (output plot to R/Rstudio)*

print(multi.plot)

pdf('multi.pdf', width = 28, height = 18)

print(multi.plot)

dev.off()

*####Interactions in the full model*

glmulti(A_per_HH~age_brackets+ Discard_Sellby+Discard_veg+Fussy+

HHSize+job_new+Local.authority+Q31_Recoded+

Discard_veg:Fussy+Discard_veg:HHSize+Discard_veg:job_new +Discard_veg:Local.authority+

Fussy:HHSize+Fussy:job_new+Fussy:Local.authority+

HHSize:job_new+Fussy:Local.authority+

job_new:Local.authority,data=data1,level=1, method='d',confsetsize=100,plotty=T)

*#303227658*

*#256*

fitI<-glm(A_per_HH~age_brackets+ Discard_Sellby+Discard_veg+Fussy+

HHSize+job_new+Local.authority+Q31_Recoded+

Discard_veg:Fussy+Discard_veg:HHSize+Discard_veg:job_new +Discard_veg:Local.authority+

Fussy:HHSize+Fussy:job_new+Fussy:Local.authority+

HHSize:job_new+Fussy:Local.authority+

job_new:Local.authority,data=data1)

dd<-dredge(fitI)

ds<-subset(dd,delta<2)

finalI<-model.avg(ds)

summary(finalI)

*##############remove discard*

names(data)

drops <- c("Discard_fruit", "Discard_cooked","Discard_meal", "Discard_unused", "Discard_packs","Discard_mould", "Discard_Sellby", "Discard_veg", "Discard_Bread", "Discard_meat",

"Discard_cheese", "Discard_milk", "Discard_ready", "Discard_home","Discard_parts" )

data2<-data[ , !(names(data) %in% drops)]

names(data2)

glmulti(A_per_HH~.,data=data2,level=1, method='d',confsetsize=10,plotty=T)

*#Error too many*

glmulti(A_per_HH~.,data=data2,level=2, method='d',confsetsize=10,plotty=T)*#with interaction*

*#Error too many*

Boruta2 <- Boruta(A_per_HH ~ ., data=data2, doTrace = 2, ntree = 500)

print(Boruta2)

plot(Boruta2)

plot(Boruta2, xlab = "", xaxt = "n")

lz<-lapply(1:ncol(Boruta2$ImpHistory),**function**(i)

Boruta2$ImpHistory[is.finite(Boruta2$ImpHistory[,i]),i])

names(lz) <- colnames(Boruta2$ImpHistory)

Labels <- sort(sapply(lz,median))

axis(side = 1,las=2,labels = names(Labels),

at = 1:ncol(Boruta2$ImpHistory), cex.axis = 0.55)

myvars<-c("A_per_HH","job_new","Local.authority", "Fussy",

"HHSize", "Q33_Recoded", "Q31_Recoded",

"children_3_to_11","CupboardTinned","CupboardFish",

"CupboardMeat","age_brackets","CupboardSalad",

"CupboardBread","CupboardFrozen","CupboardFruit")

data2<-data[myvars]

glmulti(A_per_HH~.,data=data2,level=1, method='d',confsetsize=10,plotty=T)

*#32768*

glmulti(A_per_HH~.,data=data2,level=2, method='d',confsetsize=10,plotty=T)*#with interaction*

*#1e9*

*#model selection*

fit2<-glm(A_per_HH~.,data=data2)

dd2<-dredge(fit2)

ds2<-subset(dd2,delta<2)

ds2

summary(fit2)

*###interaction*

glmulti(A_per_HH~Fussy+job_new+Local.authority+HHSize+Q31_Recoded+age_brackets,data=data2,level=2, method='d',confsetsize=10,plotty=T)*#with interaction*

*#47850*

glmulti(A_per_HH~Fussy+job_new+Local.authority+HHSize+Q31_Recoded+age_brackets,data=data2,level=2, method='h',confsetsize=10,plotty=T)*#with interaction*

modI<-glm(A_per_HH ~ job_new + Local.authority + HHSize + Q31_Recoded +

age_brackets + Fussy + age_brackets:job_new + job_new:Fussy +

Local.authority:Fussy,data=data2)

modII<-glm(A_per_HH ~ job_new + Local.authority + HHSize + Q31_Recoded +

age_brackets + Fussy + age_brackets:job_new + job_new:Fussy +

Local.authority:Fussy + age_brackets:Fussy,data=data2)

sum<-model.avg(modI,modII)

summary(sum)

*##############remove LA*

names(data)

myvars<-c("A_per_HH", "Q36_Recoded",

"job_new","Q31_Recoded",

"Fussy", "HHSize",

"Q33_Recoded", "children_3_to_11",

"age_brackets","CupboardMeat",

"Discard_Sellby", "Discard_packs", "Discard_cheese","Discard_veg",

"CupboardMeat")

data3<-data[myvars]

Boruta4 <- Boruta(A_per_HH ~ ., data=data3, doTrace = 2, ntree = 500)

print(Boruta4)

plot(Boruta4)

plot(Boruta4, xlab = "", xaxt = "n")

lz<-lapply(1:ncol(Boruta4$ImpHistory),**function**(i)

Boruta4$ImpHistory[is.finite(Boruta4$ImpHistory[,i]),i])

names(lz) <- colnames(Boruta4$ImpHistory)

Labels <- sort(sapply(lz,median))

axis(side = 1,las=2,labels = names(Labels),

at = 1:ncol(Boruta4$ImpHistory), cex.axis = 0.55)

*#model selection*

glmulti(A_per_HH~.,data=data3,level=1, method='d',confsetsize=10,plotty=T)

*#16384*

glmulti(A_per_HH~.,data=data3,level=2, method='d',confsetsize=10,plotty=T)*#with interaction*

*#1e9*

*#model selection*

fit3<-glm(A_per_HH~.,data=data3)

dd3<-dredge(fit3)

ds3<-subset(dd3,delta<2)

ds3

mod.avg(ds3)

*##interactions*

glmulti(A_per_HH~HHSize+job_new+Q31_Recoded+children_3_to_11,data=data3,level=2, method='h',confsetsize=10,plotty=T)

*#113*

fit3I<-glm(A_per_HH~HHSize+job_new+Q31_Recoded+children_3_to_11+

children_3_to_11:HHSize +children_3_to_11:job_new + children_3_to_11:Q31_Recoded,data=data3)

dd3I<-dredge(fit3I)

ds3I<-subset(dd3I,delta<2)

ds3I

sum<-model.avg(ds3I)

summary(sum)

*####*

*##########remove LA & Discard*

names(data)

drops <- c("Local.authority","Discard_fruit",

"Discard_cooked","Discard_meal",

"Discard_unused", "Discard_packs",

"Discard_mould", "Discard_Sellby",

"Discard_veg", "Discard_Bread",

"Discard_meat","Discard_cheese", "Discard_milk",

"Discard_ready", "Discard_home","Discard_parts" )

data4<-data[ , !(names(data) %in% drops)]

*#model selection*

*#model selection*

myvars<-c("A_per_HH","Fussy","HHSize",

"CupboardVeg",

"CupboardBread",

"CupboardMeat",

"CupboardFish",

"CupboardMilk",

"CupboardTinned",

"CupboardFrozen",

"CupboardSalad",

"age_brackets",

"Q33_Recoded",

"Q31_Recoded",

"Q36_Recoded",

"job_new", "children_3_to_11")

data4<-data[myvars]

glmulti(A_per_HH~HHSize+.,data=data4,level=1, method='d',confsetsize=10,plotty=T)

*#65536*

glmulti(A_per_HH~.,data=data4,level=2, method='d',confsetsize=10,plotty=T)*#with interaction*

*#1e9*

fit4<-glm(A_per_HH~.,data=data4)

dd4<-dredge(fit4)

ds4<-subset(dd4,delta<2)

ds4

summary(ds4)

model1<-glm(A_per_HH~age_brackets+Fussy+HHSize+job_new+Q31_Recoded, data=data4)

model2<-glm(A_per_HH~age_brackets+CupboardVeg+Fussy+HHSize+job_new+Q31_Recoded, data=data4)

model3<-glm(A_per_HH~age_brackets+children_3_to_11+Fussy+HHSize+job_new+Q31_Recoded, data=data4)

m4<-model.avg(model1,model2,model3)

summary(m4)

*#plot Figure 1 Random forests*

*# wrap1$Foodwaste<-wrap1$A_per_HH*

*# wrap1$A_per_HH<-NULL*

*# wrap1$Household<-wrap$HHSize*

*# wrap1$HHSize<-NULL*

*# wrap1$Vegetables<-wrap1$Discard_veg*

*# wrap1$Composition<-wrap1$Q33_Recoded*

*# wrap1$Ownership<-wrap1$Q31_Recoded*

*# wrap1$Housetype<-wrap1$Q36_Recoded*

*#*

*# wrap$Ownership<-wrap$Q31_Recoded*

*# wrap$Housetype<-wrap$Q36_Recoded*

par(mfrow = c(2,2))

plot(Boruta1, xlab = "", xaxt = "n", main="A")

lz<-lapply(1:ncol(Boruta1$ImpHistory),**function**(i)

Boruta1$ImpHistory[is.finite(Boruta1$ImpHistory[,i]),i])

colnames(Boruta1$ImpHistory)<-c("Local.authority", "Fussy", "HHSize", "ShoppingTrips",

"LeftOvers", "RiceLeft", "RiceLeft2", "PastaLeft" ,

"PastaLeft2", "Discard_fruit","Discard_cooked", "Discard_meal" ,

"Discard_unused", "Discard_packs","Discard_mould" , "Discard_Sellby" ,

"Discard_veg", "Discard_Bread","Discard_meat" , "Discard_cheese" ,

"Discard_milk", "Discard_ready","Discard_home" , "Discard_parts" ,

"HowDisposed", "ShoppingHabits","Preplan" , "CupboardFruit" ,

"CupboardVeg", "CupboardBread","CupboardMeat" , "CupboardFish" ,

"CupboardMilk", "CupboardReady","CupboardTinned" , "CupboardFrozen",

"CupboardSalad", "PreplanList","Cheese" , "Apple" ,

"Carrot", "gender","age_brackets" , "Age_over65" ,

"Ownership", "Housetype","Composition", "children_3_to_11",

"job_new", "social_grouping","shadowMax","shadowMean","shadowMin" )

names(lz) <- colnames(Boruta1$ImpHistory)

Labels <- sort(sapply(lz,median))

axis(side = 1,las=2,labels = names(Labels),

at = 1:ncol(Boruta1$ImpHistory), cex.axis = 0.7)

plot(Boruta2, xlab = "", xaxt = "n", main="B")

lz<-lapply(1:ncol(Boruta2$ImpHistory),**function**(i)

Boruta2$ImpHistory[is.finite(Boruta2$ImpHistory[,i]),i])

colnames(Boruta2$ImpHistory)<-c("Local.authority", "Fussy", "HHSize", "ShoppingTrips",

"LeftOvers", "RiceLeft", "RiceLeft2", "PastaLeft" ,

"PastaLeft2", "HowDisposed", "ShoppingHabits","Preplan" , "CupboardFruit" ,

"CupboardVeg", "CupboardBread","CupboardMeat" , "CupboardFish" ,

"CupboardMilk", "CupboardReady","CupboardTinned" , "CupboardFrozen",

"CupboardSalad", "PreplanList","Cheese" , "Apple" ,

"Carrot", "gender","age_brackets" , "Age_over65" ,

"Ownership", "Housetype","Composition", "children_3_to_11",

"job_new", "social_grouping","shadowMax","shadowMean","shadowMin" )

names(lz) <- colnames(Boruta2$ImpHistory)

Labels <- sort(sapply(lz,median))

axis(side = 1,las=2,labels = names(Labels),

at = 1:ncol(Boruta2$ImpHistory), cex.axis = 0.7)

*#Boruta3 <- Boruta(A_per_HH ~ ., data=data4, doTrace = 2, ntree = 500)*

*#print(Boruta3)*

*#plot(Boruta3)*

plot(Boruta3, xlab = "", xaxt = "n", main="C")

lz<-lapply(1:ncol(Boruta3$ImpHistory),**function**(i)

Boruta3$ImpHistory[is.finite(Boruta3$ImpHistory[,i]),i])

colnames(Boruta3$ImpHistory)<-c("Fussy", "HHSize", "CupboardVeg", "CupboardBread","CupboardMeat" , "CupboardFish" ,

"CupboardMilk", "CupboardTinned" , "CupboardFrozen",

"CupboardSalad", "age_brackets" , "Ownership", "Housetype","Composition","job_new",

"children_3_to_11","shadowMax","shadowMean","shadowMin" )

names(lz) <- colnames(Boruta3$ImpHistory)

Labels <- sort(sapply(lz,median))

axis(side = 1,las=2,labels = names(Labels),

at = 1:ncol(Boruta3$ImpHistory), cex.axis = 0.55)

plot(Boruta4, xlab = "", xaxt = "n", main="D")

lz<-lapply(1:ncol(Boruta4$ImpHistory),**function**(i)

Boruta4$ImpHistory[is.finite(Boruta4$ImpHistory[,i]),i])

colnames(Boruta4$ImpHistory)<-c("Housetype", "job_new", "Ownership", "Fussy", "HHSize", "Composition",

"children_3_to_11", "age_brackets", "CupboardMeat", "Discard_Sellby",

"Discard_packs","Discard_cheese", "Discard_veg",

"shadowMax","shadowMean","shadowMin" )

names(lz) <- colnames(Boruta4$ImpHistory)

Labels <- sort(sapply(lz,median))

axis(side = 1,las=2,labels = names(Labels),

at = 1:ncol(Boruta4$ImpHistory), cex.axis = 0.55)
