# Supplementary material for: Model selection and averaging in the assessment of the drivers of household food waste to reduce the probability of false positives
Source: PLoS One. 2018 Feb 1;13(2):e0192075. doi: 10.1371/journal.pone.0192075 (PMC5794155; doi:10.1371/journal.pone.0192075)
Supplement: S1 Table — The variables are sorted by z value. (DOCX) [file pone.0192075.s002.docx]

**S1 Table**

**S1 Table. Model average coefficients for the full model including interaction terms. The variables are sorted by z value.**

|  | Estimate | Std.Error | Adjusted SE | z value |
| --- | --- | --- | --- | --- |
| HHSize5 | 1442.29 | 214.79 | 214.94 | 6.71 |
| HHSize4 | 1100.37 | 167.72 | 167.84 | 6.56 |
| HHSize3 | 985.03 | 162.25 | 162.37 | 6.07 |
| HHSize6 | 1415.73 | 251.65 | 251.83 | 5.62 |
| Fussy | 7658.90 | 1949.36 | 1950.74 | 3.93 |
| Discard_vegA_small_amount:Fussy | -6350.51 | 1883.20 | 1884.53 | 3.37 |
| Discard_vegNone:Fussy | -5886.42 | 1820.33 | 1821.62 | 3.23 |
| Local.authority5 | -766.75 | 253.12 | 253.30 | 3.03 |
| HHSize2 | 388.92 | 129.25 | 129.35 | 3.01 |
| Discard_vegQuite_a_lot:Fussy | -9427.95 | 3154.49 | 3156.73 | 2.99 |
| (Intercept) | 1969.88 | 690.54 | 691.02 | 2.85 |
| Discard_vegHardly_any:Fussy | -5137.02 | 1848.25 | 1849.55 | 2.78 |
| Local.authority4 | -552.89 | 206.46 | 206.60 | 2.68 |
| Discard_vegDon't_eat_it:Fussy | -5549.94 | 2125.17 | 2126.68 | 2.61 |
| Fussy:job_newpaid_work | -1917.06 | 789.62 | 790.17 | 2.43 |
| Fussy:job_newnot_working_(other_reasons) | -2029.02 | 925.22 | 925.86 | 2.19 |
| Local.authority1 | 512.12 | 240.02 | 240.19 | 2.13 |
| Local.authority3 | -402.68 | 192.41 | 192.55 | 2.09 |
| Local.authority2 | -397.63 | 204.88 | 205.03 | 1.94 |
| Q31_RecodedOwned_outright | -388.55 | 222.03 | 222.08 | 1.75 |
| Discard_vegSome:Fussy | -3401.28 | 2040.74 | 2042.17 | 1.67 |
| Q31_RecodedOwned_with_mortgage | -294.94 | 192.07 | 192.14 | 1.54 |
| Fussy:job_newretired | -1177.92 | 816.16 | 816.73 | 1.44 |
| Local.authority6 | -307.58 | 218.42 | 218.58 | 1.41 |
| job_newretired | -252.67 | 205.79 | 205.94 | 1.23 |
| Q31_RecodedPrivate_rent | -228.70 | 187.71 | 187.80 | 1.22 |
| Local.authority7 | -220.21 | 206.57 | 206.72 | 1.07 |
| Discard_vegHardly_any | -530.64 | 530.08 | 530.45 | 1.00 |
| job_newpaid_work | 189.41 | 204.41 | 204.55 | 0.93 |
| age_brackets35_64 | -114.70 | 134.22 | 134.27 | 0.85 |
| Discard_vegNone | -419.69 | 521.95 | 522.32 | 0.80 |
| Q31_RecodedOther_Don't_know | -294.16 | 370.40 | 370.63 | 0.79 |
| Local.authority8 | -135.75 | 185.63 | 185.77 | 0.73 |
| Discard_vegDon't_eat_it | -418.36 | 624.36 | 624.80 | 0.67 |
| Discard_SellbyQuite_a_lot | 488.32 | 864.79 | 865.34 | 0.56 |
| Discard_vegSome | -314.94 | 563.63 | 564.03 | 0.56 |
| Local.authority9 | -100.69 | 207.89 | 208.04 | 0.48 |
| Discard_vegA_small_amount | -249.90 | 532.32 | 532.70 | 0.47 |
| Discard_SellbyA_small_amount | 229.32 | 524.56 | 524.91 | 0.44 |
| Local.authority10 | 82.49 | 211.98 | 212.13 | 0.39 |
| Discard_SellbyDon't_eat_it | -213.31 | 595.40 | 595.80 | 0.36 |
| Discard_SellbyNone | -138.01 | 501.48 | 501.83 | 0.28 |
| Local.authority11 | 39.54 | 188.26 | 188.39 | 0.21 |
| job_newnot_working_(other_reasons) | -55.78 | 273.95 | 274.14 | 0.20 |
| Discard_SellbyHardly_any | 53.00 | 502.77 | 503.12 | 0.11 |
| Discard_vegQuite_a_lot | 35.99 | 944.54 | 945.21 | 0.04 |
| Discard_SellbySome | 15.18 | 521.54 | 521.91 | 0.03 |
