# Supplementary material for: Model selection and averaging in the assessment of the drivers of household food waste to reduce the probability of false positives
Source: PLoS One. 2018 Feb 1;13(2):e0192075. doi: 10.1371/journal.pone.0192075 (PMC5794155; doi:10.1371/journal.pone.0192075)
Supplement: S2 Table — (DOCX) [file pone.0192075.s003.docx]

**S2 Table**

**S2 Table. Selected models and averaged coefficients (sorted by z value) for the model with discard behaviour variables excluded**.

| Component models: | df | logLik | AICc | delta | weight |
| --- | --- | --- | --- | --- | --- |
| Avoidable waste per Household ~ age+Fussy eaters+Household size+Employment+Local authority+Home ownership+age:Employment+Fussy:Employment | 44 | -15494.69 | 31079.72 | 0 | 0.55 |
| Avoidable waste per Household ~ age+Fussy eaters+Household size+Employment+Local authority+Home ownership+age:Fussy+age:Employment+Fussy:Employment | 45 | -15493.85 | 31080.14 | 0.42 | 0.45 |
|  |  |  |  |  |  |
|  |  |  |  |  |  |
|  |  |  |  |  |  |
|  | Estimate | Std.Error | AdjustedSE | z value |  |
| HHSize4 | 1210.38 | 168.3 | 168.42 | 7.187 |  |
| HHSize5 | 1422.9 | 217.46 | 217.61 | 6.539 |  |
| HHSize3 | 967.23 | 164.75 | 164.87 | 5.867 |  |
| HHSize6 | 1371.26 | 254.53 | 254.71 | 5.384 |  |
| Q31_RecodedOwned_outright | -473.83 | 148.35 | 148.45 | 3.192 |  |
| Fussy:job_newpaid_work | -2535.75 | 830.01 | 830.6 | 3.053 |  |
| job_newpaid_work | 1727.13 | 597.59 | 598.02 | 2.888 |  |
| HHSize2 | 374.74 | 129.95 | 130.04 | 2.882 |  |
| age_brackets35_64:job_newnot_working_(other_reasons) | -1941.37 | 681.15 | 681.64 | 2.848 |  |
| Local.authority4 | -659.56 | 235.13 | 235.3 | 2.803 |  |
| age_brackets35_64:job_newpaid_work | -1686.51 | 615.09 | 615.52 | 2.74 |  |
| job_newnot_working_(other_reasons) | 1590.4 | 651.56 | 652.02 | 2.439 |  |
| Fussy:job_newnot_working_(other_reasons) | -2350.61 | 973.53 | 974.22 | 2.413 |  |
| Q31_RecodedOwned_with_mortgage | -352.21 | 151.41 | 151.52 | 2.325 |  |
| age_brackets35_64 | 1330.31 | 609.23 | 609.66 | 2.182 |  |
| Fussy | 2344.7 | 1076.57 | 1077.28 | 2.176 |  |
| Local.authority5 | -602.37 | 288.3 | 288.51 | 2.088 |  |
| Fussy:job_newretired | -1771.81 | 853.06 | 853.66 | 2.076 |  |
| Local.authority2 | -453.21 | 231.54 | 231.71 | 1.956 |  |
| Fussy:Local.authority1 | 1996.13 | 1029.09 | 1029.82 | 1.938 |  |
| Q31_RecodedPrivate_rent | -324.14 | 172.77 | 172.89 | 1.875 |  |
| Local.authority3 | -348.45 | 221.16 | 221.31 | 1.574 |  |
| Fussy:Local.authority11 | -1157.37 | 809.78 | 810.35 | 1.428 |  |
| Fussy:Local.authority8 | 1064.66 | 820.01 | 820.59 | 1.297 |  |
| Fussy:Local.authority2 | 1284.22 | 997.14 | 997.85 | 1.287 |  |
| Q31_RecodedOther_Don't_know | -477.79 | 382.85 | 383.12 | 1.247 |  |
| Fussy:Local.authority6 | -1078.26 | 879.35 | 879.97 | 1.225 |  |
| Local.authority8 | -253.56 | 213.66 | 213.81 | 1.186 |  |
| Fussy:Local.authority7 | -848.09 | 895.69 | 896.33 | 0.946 |  |
| Local.authority9 | -193.81 | 237.96 | 238.13 | 0.814 |  |
| Fussy:Local.authority9 | 668.39 | 859.72 | 860.33 | 0.777 |  |
| Fussy:Local.authority5 | -823.02 | 1059.66 | 1060.41 | 0.776 |  |
| Local.authority11 | 134.28 | 213.44 | 213.59 | 0.629 |  |
| age_brackets35_64:Fussy | 297.85 | 480.04 | 480.22 | 0.62 |  |
| Local.authority6 | -148.3 | 247.11 | 247.29 | 0.6 |  |
| Local.authority1 | 175.68 | 303.51 | 303.72 | 0.578 |  |
| Local.authority10 | 133.35 | 246.18 | 246.35 | 0.541 |  |
| Fussy:Local.authority3 | -416.84 | 774.89 | 775.44 | 0.538 |  |
| Local.authority7 | -124.45 | 235.07 | 235.23 | 0.529 |  |
| (Intercept) | 215.2 | 627.28 | 627.72 | 0.343 |  |
| Fussy:Local.authority4 | 259.64 | 855.9 | 856.51 | 0.303 |  |
| job_newretired | -393.71 | 1377.51 | 1378.49 | 0.286 |  |
| Fussy:Local.authority10 | -184.85 | 829.3 | 829.89 | 0.223 |  |
| age_brackets35_64:job_newretired | 26.32 | 1383.56 | 1384.54 | 0.019 |  |
