# Supplementary material for: Model selection and averaging in the assessment of the drivers of household food waste to reduce the probability of false positives
Source: PLoS One. 2018 Feb 1;13(2):e0192075. doi: 10.1371/journal.pone.0192075 (PMC5794155; doi:10.1371/journal.pone.0192075)
Supplement: S3 Table — (DOCX) [file pone.0192075.s004.docx]

**S3 Table**

**S3 Table. Selected models and averaged coefficients (sorted by z value) for the model with local authority excluded.**

| Component models: | df | logLik | AICc | delta | weight |
| --- | --- | --- | --- | --- | --- |
| Avoidable waste per Household ~ Children 3 to 11+ Household size+ Employment+ Household ownership+ Children 3 to 11: Household size+ Children 3 to 11: Employment | 22 | -15534.8 | 31114.1 | 0 | 0.212 |
| Avoidable waste per Household ~ Children 3 to 11+ Household size+ Employment+ Household ownership+ Children 3 to 11: Household size | 18 | -15539 | 31114.4 | 0.26 | 0.186 |
| Avoidable waste per Household ~ Children 3 to 11+ Household size+ Employment | 14 | -15543.1 | 31114.5 | 0.35 | 0.178 |
| Avoidable waste per Household ~ Children 3 to 11+ Household size+ Employment+ Household ownership+ Children 3 to 11: Household size+ Children 3 to 11: Employment+Children 3 to 11: Home ownership | 26 | -15531 | 31114.8 | 0.7 | 0.149 |
| Avoidable waste per Household ~ Children 3 to 11+ Household size+ Employment+ Household ownership+ Children 3 to 11: Household size+Children 3 to 11: Home ownership | 22 | -15535.4 | 31115.3 | 1.22 | 0.115 |
| Avoidable waste per Household ~ Children 3 to 11+ Household size+ Employment+ Household ownership | 15 | -15542.9 | 31116 | 1.9 | 0.082 |
| Avoidable waste per Household ~ Children 3 to 11+ Household size+ Employment+ Household ownership+ Children 3 to 11: Household size | 19 | -15538.8 | 31116.1 | 1.99 | 0.078 |
|  |  |  |  |  |  |
|  | Estimate | Std.Error | Adjusted SE | z value |  |
| HHSize4 | 1322.68 | 202.67 | 202.80 | 6.52 |  |
| HHSize3 | 1029.57 | 175.58 | 175.70 | 5.86 |  |
| (Intercept) | 1389.81 | 263.25 | 263.40 | 5.28 |  |
| HHSize5 | 1495.79 | 284.90 | 285.06 | 5.25 |  |
| Q31_RecodedOwned_outright | -503.67 | 158.49 | 158.59 | 3.18 |  |
| HHSize2 | 394.67 | 131.30 | 131.40 | 3.00 |  |
| Q31_RecodedOwned_with_mortgage | -431.48 | 159.77 | 159.88 | 2.70 |  |
| job_newretired | -314.05 | 227.04 | 227.17 | 1.38 |  |
| HHSize6 | 951.85 | 782.52 | 782.72 | 1.22 |  |
| children_3_to_11yes | 1388.22 | 1158.99 | 1159.20 | 1.20 |  |
| children_3_to_11yes:job_newretired | -1372.44 | 1180.96 | 1181.21 | 1.16 |  |
| children_3_to_11yes:job_newpaid_work | -657.26 | 598.92 | 599.09 | 1.10 |  |
| children_3_to_11yes:job_newnot_working_(other_reasons) | -671.59 | 651.22 | 651.43 | 1.03 |  |
| Q31_RecodedOther_Don't_know | -400.02 | 393.00 | 393.28 | 1.02 |  |
| job_newnot_working_(other_reasons) | -289.21 | 288.01 | 288.19 | 1.00 |  |
| Q31_RecodedPrivate_rent | -226.63 | 234.56 | 234.66 | 0.97 |  |
| children_3_to_11yes:HHSize4 | -718.62 | 936.08 | 936.25 | 0.77 |  |
| children_3_to_11yes:HHSize2 | -820.80 | 1074.84 | 1075.04 | 0.76 |  |
| children_3_to_11yes:HHSize3 | -559.17 | 786.88 | 787.08 | 0.71 |  |
| children_3_to_11yes:HHSize5 | -435.56 | 701.58 | 701.83 | 0.62 |  |
| children_3_to_11yes:Q31_RecodedPrivate_rent | -204.27 | 386.59 | 386.65 | 0.53 |  |
| children_3_to_11yes:Q31_RecodedOwned_outright | -135.31 | 303.37 | 303.47 | 0.45 |  |
| job_newpaid_work | 77.82 | 231.46 | 231.59 | 0.34 |  |
| children_3_to_11yes:Q31_RecodedOther_Don't_know | 130.84 | 624.86 | 625.24 | 0.21 |  |
| children_3_to_11yes:Q31_RecodedOwned_with_mortgage | -28.41 | 169.90 | 170.01 | 0.17 |  |
| children_3_to_11yes:HHSize6 | 0.00 | 0.00 | 0.00 | NA |  |
