# Supplementary material for: Model selection and averaging in the assessment of the drivers of household food waste to reduce the probability of false positives
Source: PLoS One. 2018 Feb 1;13(2):e0192075. doi: 10.1371/journal.pone.0192075 (PMC5794155; doi:10.1371/journal.pone.0192075)
Supplement: S4 Table — (DOCX) [file pone.0192075.s005.docx]

**S4 Table**

**S4 Table. Selected models and averaged coefficients (sorted by z value) for the model with local authority and discard behaviours excluded.**

| Component models: | df | logLik | AICc | delta | weight |
| --- | --- | --- | --- | --- | --- |
| Avoidable waste per Household ~ age+Fussy eaters+ Household size+ Employment+ Household ownership | 16 | -15537 | 31107.2 | 0 | 0.54 |
| Avoidable waste per Household ~ age+Check cupboard for veg+Fussy eaters+ Household size+ Employment+ Household ownership | 19 | -15535 | 31108.7 | 1.55 | 0.25 |
| Avoidable waste per Household ~ age+Children 3 to 11+Fussy eaters+ Household size+ Employment+ Household ownership | 17 | -15537 | 31109.1 | 1.95 | 0.21 |
|  |  |  |  |  |  |
|  | Estimate | Std.Error | Adjusted SE | z value |  |
| HHSize4 | 1252.25 | 171.38 | 171.50 | 7.30 |  |
| HHSize5 | 1585.09 | 219.50 | 219.65 | 7.22 |  |
| HHSize3 | 1038.15 | 165.88 | 166.00 | 6.25 |  |
| HHSize6 | 1491.85 | 258.92 | 259.10 | 5.76 |  |
| (Intercept) | 1913.98 | 524.49 | 524.66 | 3.65 |  |
| HHSize2 | 409.27 | 130.52 | 130.62 | 3.13 |  |
| Q31_RecodedOwned_outright | -461.37 | 147.27 | 147.38 | 3.13 |  |
| Fussy | 475.39 | 185.19 | 185.32 | 2.57 |  |
| Q31_RecodedOwned_with_mortgage | -375.17 | 150.29 | 150.40 | 2.50 |  |
| job_newretired | -455.90 | 193.13 | 193.26 | 2.36 |  |
| Q31_RecodedPrivate_rent | -359.22 | 172.91 | 173.03 | 2.08 |  |
| age_brackets35_64 | -254.54 | 125.55 | 125.64 | 2.03 |  |
| job_newnot_working_(other_reasons) | -461.96 | 239.98 | 240.15 | 1.92 |  |
| Q31_RecodedOther_Don't_know | -419.61 | 383.74 | 384.00 | 1.09 |  |
| job_newpaid_work | -111.72 | 190.15 | 190.28 | 0.59 |  |
| CupboardVegYes | -232.67 | 484.73 | 484.84 | 0.48 |  |
| CupboardVegNo | -189.57 | 428.63 | 428.76 | 0.44 |  |
| CupboardVegDontKnow | -217.16 | 524.11 | 524.29 | 0.41 |  |
| children_3_to_11yes | 8.12 | 62.25 | 62.30 | 0.13 |  |
